# Atlanta Medical College

**Published:** 1868

**Authors:** 


### ATLANTA MEDICAL COLLEGE.

The regular session of this institution will commence on the first Monday in May next. Arrangements have been made for a large class, and there is scarcely a doubt that our expectations will be realized. We have received letters from every Southern State, conveying the most gratifying assurances on this subject, and from this and other sources we incline to the opinion that the class in number will fall little, if any, below those before the war.

The Atlanta Medical College offers every inducement that any other institution can offer to gentlemen seeking a medical education. Prof. Armstrong is now in Europe perfecting himself in the study of microscopy, which will constitute a part of the course, thereby enabling the students to become familiar with the instrument, so as to observe the capillary circulation, and the healthy and morbid condition of the minute structures, etc., etc.

Steps have been taken by the Faculty to admit two beneficiaries from each Congressional District throughout the Southern States, from amongst those who are too poor to pay full charges, upon the payment of twenty dollars to cover College expenses. Certificates of good standing and also the pecuniary condition of the applicant to accompany the application.

Facilities for practical study are ample. There is on the College grounds a hospital numbering one hundred patients, embracing nearly all the varieties of known disease, besides the College Dispensary, in which ten to twenty are examined daily. It will thus be seen that no place can offer inducements superior to Atlanta in this respect. This city is celebrated for its fine water, and its salubrious atmosphere. No place on the globe is healthier.

